# Is nutritional support needed in late preterm infants?

**DOI:** 10.1186/s12887-015-0511-8

**Published:** 2015-11-23

**Authors:** Maria Lorella Giannì, Paola Roggero, Pasqua Piemontese, Nadia Liotto, Anna Orsi, Orsola Amato, Francesca Taroni, Laura Morlacchi, Dario Consonni, Fabio Mosca

**Affiliations:** Department of Clinical Science and Community Health, Neonatal Intensive Care Unit, Fondazione I.R.C.C.S. Ca’ Granda Ospedale Maggiore Policlinico, University of Milan, via Commenda 12, 20122 Milano, Italy; Fondazione IRCCS Ca’ Granda Ospedale Maggiore Policlinico, Epidemiology Unit, Via San Barnaba 8, 20122 Milan, Italy

**Keywords:** Late preterm infants, Nutritional support, Feeding issues

## Abstract

**Background:**

Late preterm birth accounts for 70 % of all preterm births. While the impact of feeding problems in very preterm infants has been widely investigated, data on late preterm infants’ feeding issues are scarce. The aim of the present study was to investigate the need of nutritional support during hospital stay in a cohort of late preterm infants and to identify the factors that most contribute to its occurrence.

**Methods:**

We analyzed the medical records of late preterm infants, born 2011–2013, admitted to a single institution. Neonatal data, the need for nutritional support, defined as the need for parenteral nutrition or intravenous fluids or tube feeding, and the feeding status at discharge were retrieved. The occurrence of respiratory distress syndrome, congenital malformations/chromosomal diseases, cardiac diseases, sepsis, hypoglycemia, poor feeding and the need for surgical intervention were also collected.

**Results:**

A total of 1768 late preterm infants were included. Among the 592 infants requiring a nutritional support, 228 developed a respiratory distress syndrome, two developed a sepsis, one presented with a cardiac disease, 24 underwent a surgical intervention, eight had a chromosomal disease/congenital malformation, 80 had hypoglycemia. In addition, 100 infants required nutritional support due to poor feeding and 149 were born small for gestational age. Birth weight ≤2000 g (adjusted OR = 12.2, 95 % CI 7.5-19.9, *p <* 0.0001), gestational age of 34 weeks (adjusted OR = 4.08, 95 % CI 2.8-5.9, *p <* 0.0001), being small for gestational age (adjusted OR = 2.17, 95 % CI 2.8-5.9, p=0.001), having a respiratory distress syndrome (adjusted OR = 79.6, 95 % CI 47.2-134.3, *p <* 0.0001) and the need of surgical intervention (adjusted OR = 49.4, 95 % CI 13.9-174.5, *p <* 0.0001) were associated with a higher risk of need of nutritional support during hospital stay.

**Conclusions:**

Late preterm infants are at relatively high risk of requiring nutritional support during hospital stay, especially if they have a birth weight ≤2000 g, a gestational age of 34 weeks, are born small for gestational age, develop a respiratory distress syndrome and require a surgical intervention. The present findings add to the knowledge of late preterm infants’ feeding issues and may contribute to tailoring nutritional approaches for these infants.

## Background

Late preterm birth, defined as a birth that occurs between 34 0/7 and 36 6/7 week of gestation, contributes significantly to the premature rate, accounting for 70 % of all preterm births [1, 2]. Incidence of late preterm birth has markedly increased during the past two decades and has been associated with increased prevalence of medical issues [3, 4]. Feeding difficulties, related to maternal and neonatal reasons, have been reported to occur with high frequency. These difficulties definitely cause increased needs for parenteral nutrition, infusion therapy and tube feeding leading to prolonged length of stay [5, 6].

While the impact of feeding problems in very preterm infants has been widely investigated [7], there is paucity of data on late preterm infants’ feeding issues [8]. The aim of the present study was to investigate the need of nutritional support during hospital stay in a cohort of infants born late preterm and to identify the factors that most contribute to its occurrence.

## Methods

We analyzed the medical records of late preterm infants born 2011–2013, admitted to Authors’ Institution, including level I, II and III of care. The Ethics Committee of the Fondazione Istituto di Ricovero e Cura a Carattere Scientifico Cà Granda Ospedale Maggiore Policlinico approved the study. Written informed consent was obtained by parents at time of infants’ admission. Inclusion criteria was gestational age 34 0/7 to 36 6/7. Exclusion criteria were newborns transferred to other Institution.

According to our internal clinical protocol, late preterm infants with a birth weight ≥1900 g, irrespective of gestational age, were admitted to level I of care, provided that clinical conditions were stable and no nutritional support was required. Infants with a birth weight <1900 grams and/or requiring any type of nutritional support were admitted/transferred to either level II or III of care, according to their clinical conditions.

## Nutritional practices

According to our internal nutritional procedure, infants with a birth weight <1500 g qualified for parenteral nutrition. In addition, infants presenting with any clinical condition that could hinder the beginning of enteral nutrition or that could interfere with the ability to feed exclusively by mouth received either parenteral nutrition/intravenous fluids or tube feeding. Provided that infants were in stable clinical conditions, mothers were encouraged to breastfeed their infant. When human milk was unavailable or insufficient, formula feeding was started.

### Data collection procedures

The neonatal, medical and feeding data were collected from the patients’ computerized medical charts from birth to discharge. The neonatologists in charge of the infants daily fulfilled the infants’ medical charts after completing their medical visit. Data were then retrieved using a software incorporated into the computerized medical charts (Neocare, i&t Informatica e Tecnologia Srl, Italy). The recorded neonatal data were: gestational age at birth, birth weight, length and head circumference, gender, singleton or multiple pregnancy, length of stay. Gestational age (GA) was based on the last menstrual period and first-trimester ultrasonogram. The infants with a birth weight <10th or ≥10th percentile for gestational age on the basis of Fenton’s growth chart [9], were respectively classified as having a weight that was small for gestational age (SGA) or appropriate for gestational age (AGA). A record was made of the occurrence of the following comorbidities: respiratory distress syndrome, defined as the need for any respiratory support; congenital malformations and cardiac diseases, diagnosed either prenatally by ultrasound assessment and/or genetic test or after birth by physical and radiological examination; sepsis, defined as the presence of a positive blood culture; hypoglycemia defined as a plasma glucose level <45 mg/dl and any disease that needed a surgical intervention. Poor feeding due to infants’ developmental immaturity and the feeding status (exclusively human milk, any human milk or exclusively formula) at discharge were also collected.

## Statistical analysis

Descriptive data are shown as means ± standard deviation (SD) or number of observations (percentage). Comparison among groups was performed by the X ^2^ test for discrete variables or by the ANOVA or the Mann–Whitney test, when appropriate, for continuous variables. The association between gestational age at birth and the need of nutritional support during hospital stay was tested by computation of odds ratios (OR) and 95 % confidence intervals (CI). In binary logistic regression analysis, the adjusted odds ratios were first calculated for the infants who did not develop comorbidities, adjusting for the following potential confounders: GA (=34 weeks *vs* >34 weeks), birth weight (≤2000 g *vs* >2000 g), gender (male *vs* female), being twin (twin *vs* singleton), small for gestational age (SGA *vs* AGA), and the need of nutritional support (yes *vs* no) as the outcome variable. The adjusted odds ratios were then calculated for all the enrolled infants, including as potential confounders, in addition to the variables previously entered, the occurrence of respiratory distress syndrome (yes *vs* no), sepsis (yes *vs* no), cardiac disease (yes *vs* no), surgical intervention (yes *vs* no), chromosomal disease/congenital malformation (yes *vs* no), hypoglycemia (yes *vs* no) and the need of nutritional support (yes *vs* no) as the outcome variable.

For analysis, infants that had needed either parenteral nutrition, intravenous fluids and/or tube feeding were pooled together and categorized as infants needing nutritional support. Furthermore, infants were grouped according to gestational age (=34 weeks *vs* > 34 weeks), and to birth weight (≤2000 g *vs* >2000 g), due to the increased risk of developing feeding difficulties in infants born with the lowest gestational ages and birth weights [10].

All statistical analyses were performed using SPSS (SPSS, Version 12; SPSS Inc., Chicago, Ill., USA).

## Results

Out of the 1768 late preterm infants included, 1176 were admitted to level I of care, 322 were admitted to level II of care and 270 were admitted to level III of care. Basic subject characteristics are shown in Table 1. At birth infants with GA of 34 weeks were significantly lighter, shorter and had a mean head circumference value lower than infants with GA of 35 and 36 weeks. Percentage of twins was significantly higher in infants born with GA of 34 weeks as compared to infants born with GA of 35 and 36 weeks. Birth weight, length and head circumference values were significantly lower in infants with GA of 35 weeks than in infants with GA of 36 weeks. Mean hospital stay (days) of infants born with GA of 34 and 35 weeks was significantly longer than that of infants born with GA of 36 weeks (14.9 ± 12.8 versus 9.9 ± 9.5 versus 7.8 ± 9.7, *p <* 0.0001).

**Table 1:** Basic subject characteristics

	Enrolled infants (*n =* 1768)	Infants with GA = 34 (*n =* 359)	Infants with GA = 35 (*n =* 571)	Infants with GA = 36 (*n =* 838)
Birth weight (g)	2404 ± 419	2126 ± 365*	2320 ± 364^**^	2581 ± 394
Birth length (cm)	46.1 ± 2.3	44.9 ± 2.6*	45.8 ± 2.0^**^	46.7 ± 2.18
Birth head circumference (cm)	32.7 ± 1.5	32.0 ± 1.8*	32.5 ± 1.4^**^	33.0 ± 1.4
SGA n(%)	451 (25.5)	106 (29.5)	149 (25.6)	199 (23.7)
Males n (%)	869 (49.2)	179 (49.9)	278 (48.7)	412 (49.2)
Twins n (%)	468 (26.5)	128 (35.7)*	163 (28.5)	177 (21.1)

*p < 0.0001 infants with GA = 34 weeks versus infants with 35 and 36 weeks

**p < 0.0001 infants with GA = 35 weeks versus infants with GA = 36 weeks

Of the enrolled infants, 14 % developed a respiratory distress syndrome (28.4 % of infants born with GA of 34 versus 12.3 % of infants born with GA of 35 versus 8.9 % of infants born with GA of 36, *p <* 0.0001), 1.5 % needed a surgery treatment, 0.7 % had a chromosomal and/or a congenital disease, 0.1 % developed a sepsis.

At discharge 63.3 % of the infants were fed any human milk (60 %, 61 % and 69 % of infants born with GA of 34, 35 and 36 weeks, respectively) and 18 % were fed exclusive human milk (12.8 %, 15.9 % and 26 % of infants born with GA of 34, 35 and 36 weeks, respectively).

Need of nutritional support was found in 592 infants. Out of the patients requiring nutritional support, 228 developed respiratory distress syndrome, two developed sepsis, one presented with a cardiac disease, 24 underwent a surgical intervention, eight had a chromosomal disease/congenital malformation, 80 had hypoglycemia. In addition, 100 infants required nutritional support due to poor feeding and 149 infants were born SGA. According to our internal nutritional procedure, all the infants requiring nutritional support were admitted to either level II or III of care.

In Table 2 is shown the need of nutritional support according to GA. Infants born with GA of 34 and 35 weeks needed nutritional support in a significantly higher percentage of cases in comparison to infants born with GA of 36 weeks. Mean hospital stay (days) of infants requiring a nutritional support was longer than that of infants that did not need any nutritional support (16.7 ± 15.8 versus 6.5 ± 3.3, *p <* 0.0001). Among infants requiring nutritional support, 76 % were fed formula whereas only 24 % were fed any human milk.

**Table 2:** Need of nutritional support according to gestational age at birth

	Enrolled infants	Infants with GA = 34 (*n =* 359)	Infants with GA = 35 (*n =* 571)	Infants with GA = 36 (*n =* 838)
Need for parenteral nutrition n(%)	78 (4.4)	38 (10.6)*	27 (4.7)***	13 (1.6)
Need for intravenous fluids n(%)	598 (33.8)	245 (68.2)*	196 (34.3)***	157 (18.7)
Need for tube feeding n(%)	46 (2.6)	19 (5.3)*	16 (2.8)**	11 (1.3)

*p < 0.0001 infants with GA = 34 weeks versus infants with 35 and 36 weeks

**p = 0.04 infants with GA = 35 weeks versus infants with GA = 36 weeks

***p < 0.0001 newborns with GA = 35 weeks versus newborns with GA = 36 weeks

Regarding infants who did not develop co-morbidities, at binary logistic regression analysis, birth weight ≤2000 g, GA of 34 weeks and possibly being born SGA were independently associated with a higher risk of nutritional support during hospital stay (Table 3). When including in the analysis the infants who have developed co-morbidities, birth weight ≤2000 g (adjusted OR = 12.2, 95 % CI 7.5-19.9, *p <* 0.0001), GA of 34 weeks (adjusted OR = 4.08, 95 % CI 2.8-5.9, *p <* 0.0001), being born SGA (adjusted OR = 2.17, 95 % CI 2.8-5.9, *p =* 0.001), having developed a respiratory distress syndrome (adjusted OR = 79.6, 95 % CI 47.2-134.3, *p <* 0.0001) and having required a surgical intervention (adjusted OR = 49.4, 95 % CI 13.9-174.5, *p <* 0.0001) resulted to be independently associated with a higher risk of receiving a nutritional support during hospital stay.

**Table 3:** Variables associated with need of nutritional support during hospital stay: binary logistic regression analysis

	Adjusted odds ratio (95 % CI)	*P*
Birth weight (g) (≤2000 *vs* >2000)	12.9 (7.0-23.7)	<0.0001
Gestational age (weeks) (34 *vs* ≥35)	5.12 (3.3-7.9)	<0.0001
Small for gestational age (SGA *vs* AGA)	1.79 (0.9-3.2)	0.05
Gender (male *vs* female)	1.06 (0.7-1.5)	0.7
Twin (yes vs no)	1.08 (0.7-1.6)	0.7

## Discussion

The findings of this study indicate that the lower the birth weight and the gestational age are, the greater the risk for needing nutritional support during hospital stay. Specifically, the need of nutritional support was registered in 33.5 % of the included late preterm infants.

These findings are in agreement with previous data reported in literature. The coordinated sucking ability that allows for the provision of sufficient intake for growth by sucking feeds alone begin to develop at 34 weeks [11]. However, late preterm infants have been reported to show immature oro-buccal coordination and swallowing mechanisms, that hamper the establishment and maintenance of adequate oral feeding skills [5]. Accordingly, we have reported that 2.6 % of the enrolled late preterm infants needed tube feeding, with the highest percentage among the infants born at 34 weeks of gestational age. Raju et al. [12] have underlined that maturation is a continuous but non-linear process, leading to different trajectories of organs’ maturation. Accordingly, most late preterm infants may have a mature breathing apparatus immediately after birth whereas they still show an immature brainstem, sucking and swallowing coordination. In addition, Medoff-Cooper et al. [13] reported that the feeding behaviors of late preterm infants at 35 to 36 weeks postmenstrual age were more immature than those of early preterm infants. The authors suggested that the earlier postnatal experience with oral feeding led to more mature feeding abilities in the early preterm infants.

As a consequence, the feeding challenges that the late preterm infants commonly experience lead to a high need for parenteral nutrition and intravenous fluids and place them at risk to be delayed in their discharge to home [8, 14, 15]. Wang et al. [14] found that the occurrence of medical problems in infants born with a gestational age of 35–36 6/7 weeks was significantly higher than in infants born at term. Specifically, the authors reported that 26.7 % infants born with a gestational age of 35–36 6/7 weeks received intravenous infusions versus 5.3 % of full term infants. In the present study, a moderately higher percentage (33.8 %) of infants needed intravenous fluids whereas 4.4 % needed parenteral nutrition. This finding could be partially explained by the fact that we have enrolled also infants born at 34 weeks of gestational age, that represented the majority of infants requiring either intravenous fluids or parenteral nutrition. The mean length of hospital stay of the preterm infants enrolled in the present study resulted to be longer than what is generally accepted for term infants born by vaginal delivery (48 h) and by after a cesarean delivery (96 h) [14]. This finding probably reflects the time needed for the late preterm infants to develop adequate feeding skills, as indicated by the longer hospital stay of the late preterm infants requiring nutritional support compared with that of the late preterm infants who did not received any nutritional support. Indeed, according to the American Academy of Pediatrics, feeding competency is considered a precondition for hospital discharge [16].

At logistic regression analysis, when considering only infants who did not develop co-morbidities, birth weight ≤2000 g, GA of 34 weeks and possibly being born SGA were independently associated with a higher risk of having nutritional support during hospital stay. In addition, when including in the analysis the infants who have developed co-morbidities, not only birth weight ≤2000 g, GA of 34 weeks and being born SGA, but also having developed a respiratory distress syndrome and having required a surgical intervention resulted to be independently associated with a higher risk of receiving a nutritional support. Indeed, out of the infants requiring a nutritional support in the present study, 58 % and 25 % presented a co-morbidity and were born SGA, respectively. Being born SGA is actually a recognized risk factor for very preterm infants for having a prolonged transition period from the beginning of oral feeding to full oral feeding [17]. The great impact of specific co-morbidities on the progression to full oral feeding has been underlined by several authors [10, 18]. Hwang et al. [18] investigated the length of transition time from the initiation to completion of full oral feeding and the medical complications that could negatively interfere with the feeding progress in a cohort of 117 very preterm infants and found that the occurrence of bronchopulmonary dysplasia and necrotizing enterocolitis were the most important medical complications associated with higher postmenstrual age at full oral feeding.

Late preterm infants have been reported to be at risk for inadequate consumption of milk feedings at the breast due to both ineffective breastfeeding behaviors and delayed onset of lactation [6]. Accordingly, in our study, infants requiring a nutritional support were fed more frequently formula than any human milk. Davanzo et al. [19] found that 47 % of infants born at 32 to 36 weeks of gestational age, discharged from the NICU, received any human milk whereas, in the present study, a moderately higher percentage (63.3 %) of the enrolled infants were fed any human milk. However, this finding could be partially explained by the fact that we have enrolled infants born at 34 to 36 weeks of gestational age, admitted to either level I, II or III of care. Accordingly, Boyle et al. [15] have reported that 64.2 % out of 1146 infants born late (34–36weeks) and moderately (32–33 weeks) preterm were fed breast milk.

The present study has limitations. First, the data have been collected from a single institution. Second, a potential bias of the current study could result from the clinical and nutritional protocols used. Hence, given these selection biases, it is not possible to generalize our findings from a single centre study of a cohort of infants to all late preterm infants. Nevertheless, a strength of the study is that it addresses a relatively large number of late preterm infants.

## Conclusion

Late preterm infants are at relatively high risk of requiring nutritional support during hospital stay, especially if they have a birth weight ≤2000 g, a gestational age of 34 weeks, are born small for gestational age, develop a respiratory distress syndrome and require a surgical intervention. The present findings add to the knowledge of late preterm infants’ feeding issues and may contribute to tailoring nutritional approaches for these infants.

## Abbreviations

GA: Gestational age
AGA: Adequate for gestational age
SGA: For small for gestational age
SD: Standard deviation
OR: Odd ratio
CI: Confidence interval

## References

[1] Dong Y, Yu JL (2011). An overview of morbidity, mortality and long-term outcomeof late preterm birth. World J Pediatr.

[2] Davidoff MJ, Dias T, Damus K, Russell R, Bettegowda VR, Dolan S (2006). Changes in the gestational age distribution among U.S. singleton births: impact on rates of late preterm birth, 1992 to 2002. Semin Perinatol.

[3] Hwang SS, Barfield WD, Smith RA, Morrow B, Shapiro-Mendoza CK, Prince CB (2013). Discharge timing, outpatient follow-up, and home care of late-preterm and early-term infants. Pediatrics.

[4] Sahni R, Polin RA (2013). Physiologic underpinnings for clinical problems in moderately preterm and late preterm infants. Clin Perinatol.

[5] Cleaveland K (2010). Feeding challenges in the late preterm infant. Neonatal Netw.

[6] Meier P, Patel AL, Wright K, Engstrom JL (2013). Management of breastfeeding during and after the maternity hospitalization for late preterm infants. Clin Perinatol.

[7] Dodrill P, Donovan T, Cleghorn G, McMahon S, Davies PS (2008). Attainment of early feeding milestones in preterm neonates. J Perinatol.

[8] DeMauro SB, Patel PR, Medoff-Cooper B, Posencheg M, Abbasi S (2011). Postdischarge feeding patterns in early- and late-preterm infants. ClinPediatr (Phila).

[9] Fenton TR (2003). A new growth chart for preterm babies: Babson and Benda’s chart updated with recent data and a new format. BMC Pediatr.

[10] Jadcherla SR, Wang M, Vijayapal AS, Leuthner SR (2010). Impact of prematurity and co-morbidities on feeding milestones in neonates: a retrospective study. J Perinatol..

[11] Mizuno K, Ueda A (2003). The maturation and coordination of sucking, swallowing, and respiration in preterm infants. J Pediatr..

[12] Raju TN (2012). Developmental physiology of late and moderate prematurity. Semin Fetal Neonatal Med..

[13] Medoff-Cooper B, McGrath JM, Shults J (2002). Feeding patterns of full-term and preterm infants at forty weeks postconceptional age. J Dev Behav Pediatr..

[14] Wang ML, Dorer DJ, Fleming MP, Catlin EA (2004). Clinical outcomes of near-term infants. Pediatrics..

[15] Boyle EM, Johnson S, Manktelow B, Seaton SE, Draper ES, Smith LK (2015). Neonatal outcomes and delivery of care for infants born late preterm or moderately preterm: a prospective population- based study. Arch Dis Child Fetal Neonatal Ed.

[16] Engle WA, Tomashek KM (2007). Wallman C, Committee on Fetus and Newborn, American Academy of Pediatrics. “Late-preterm” infants: a population at risk. Pediatrics.

[17] Bache M, Pizon E, Jacobs J, Vaillant M, Lecomte A. Effects of pre-feeding oral stimulation on oral feeding in preterm infants: a randomized clinical trial. Early Hum Dev. 2014;90:125–9. 10.1016/j.earlhumdev.2013.12.011 24461572

[18] Hwang YS, Ma MC, Tseng YM, Tsai WH (2013). Associations among perinatal factors and age of achievement of full oral feeding in very preterm infants. Pediatr Neonatol.

[19] Davanzo R, Ronfani L, Brovedani P, Demarini S (2009). Breastfeeding very-low-birth weight infants at discharge: a multicentre study using WHO definitions. Paediatr Perinat Epidemiol.

